# Gamification of active travel to school: A pilot evaluation of the Beat the Street physical activity intervention

**DOI:** 10.1016/j.healthplace.2016.03.001

**Published:** 2016-05

**Authors:** Emma Coombes, Andy Jones

**Affiliations:** a Norwich Medical School, University of East Anglia, Norwich, UK; b UKCRC Centre for Diet and Activity Research (CEDAR), Institute of Public Health, Cambridge, UK

**Keywords:** Physical activity, Active travel, School, Children, Gamification

## Abstract

Beat the Street aims to get children more active by encouraging them to walk and cycle in their neighbourhood using tracking technology with a reward scheme. This pilot study evaluates the impact of Beat the Street on active travel to school in Norwich, UK. Eighty children 8–10 yrs were recruited via an intervention and control school. They wore an accelerometer for 7 days at baseline, mid-intervention and post-intervention (+20 weeks), and completed a travel diary. Physical activity overall was not higher at follow-up amongst intervention children compared to controls. However, there was a positive association between moderate-to-vigorous physical activity (MVPA) during school commute times and the number of days on which children touched a Beat the Street sensor. This equated to 3.46 min extra daily MVPA during commute times for children who touched a sensor on 14.5 days (the mean number of days), compared to those who did not engage. We also found weekly active travel increased at the intervention school (+10.0% per child) while it decreased at the control (−7.0%), p=0.056. Further work is needed to understand how improved engagement with the intervention might impact outcomes.

## 1 Introduction

Physical activity levels in children are low, with less than a quarter of English 5–15 year olds achieving the recommended 60 min of moderate-to-vigorous physical activity 7 days a week (Townsend et al., 2015). It has been suggested that a contributing factor to childhood inactivity and associated obesity is excessive use of technology, including video games (Lamboglia et al., 2013, Arango et al., 2014). However, while video gaming is seen as having a negative impact on children’s physical activity levels, a growing body of research has examined whether it is possible to use the principles by which gaming works to get children more active and thus provide part of the solution to inactivity (Boulos and Yang, 2013, Lister et al., 2014). This process is known as gamification, which is defined as the use of game design elements in non-game contexts (Deterding et al., 2011). The theory behind gamification is that if health promotion initiatives can capture the components that make games addictive, then they can be used to improve the effectiveness of interventions by also making pro-health behaviours addictive and hence more likely to be habitualised (Cugelman, 2013).

Active travel (walking or cycling for transport), has been shown to be a major contributor to overall physical activity in children, and cross-sectional studies have demonstrated that those who actively travel to school accumulate between five and thirty-seven more minutes of MVPA per day compared to those using motorised transport (Lee et al., 2008, Faulkner et al., 2009, Southward et al., 2012, Schoeppe et al., 2013). Despite this, a recent study found that only just over half of children (58%) actively travelled to school in a sample of English 10-16 year olds (Voss and Sandercock, 2010), and data suggest that the proportion of youth actively travelling is likely to decline in the absence of initiatives to increase its prevalence (Pabayo et al., 2011). Thus interventions that successfully maintain or initiate active travel behaviours are likely to provide substantial health benefits. Gamification theory suggests it should be possible to make a routine non-game activity such as active travel into a game that is engaging and fun (Cugelman, 2013). This could be done by adding elements such as earning points for walking to school or work and allowing players to compete against themselves and each other by travelling greater distances by active means.

A recent study by Walsh and Golbeck (2014) evaluated the potential of gamification to increase levels of walking. They recruited 74 adults to wear Fitbits, a personal activity monitoring device that tracked the number of steps taken in a day, and compared step totals in three experimental conditions: a control, a social interaction experience, and a social game they developed called StepCity. They found that for newer Fitbit users, the StepCity game led to users taking more steps than they did in the control condition, suggesting that gamification played a role in initiating and maintaining increased walking levels in that study. Similarly, gamification is now incorporated into many running and cycling apps where users can gain points for each mile accrued or for reaching a target time to compete against themselves or against others (Lister et al., 2014).

The focus of this study is a recently developed intervention called Beat the Street, which incorporates gamification components. Beat the Street aims to encourage residents to walk and cycle around their local environment via the use of walk tracking technology linked to a reward scheme, with the aim that the intervention will promote long term changes in healthy behaviours (Intelligent Health, 2015). Residents are issued with a smartcard that they touch on sensors known as ‘Beat Boxes’, which are installed on lampposts around the local area. Residents are awarded a point each time they touch-in at a sensor and they compete to see who can achieve the most walks over a month, with high scorers being rewarded. For example, children receive points for their school that can be used to obtain books, whereas adults are entered into a prize draw. Beat the Street attempts to engage participants by using several key gamification strategies including providing feedback on players’ performance to allow them to set goals and monitor their improvement, allowing them to compare their progress with others, and rewarding positive behaviour, all of which are components in initiating behaviour change (Cugelman, 2013).

Beat the Street was initially trialled in the UK city of Reading and has since been implemented internationally across neighbourhoods in London, New York, Shanghai, and Vancouver. The intervention has not been formally evaluated with the exception of one recent mixed-methods study by Hunter et al. (2015), which examined whether Beat the Street increased children’s walking to and from school in cities in the UK and Canada but found somewhat mixed findings. From self-report Hunter et al. (2015) found that 97% of children reported post-intervention that walking to school helped them stay healthy, feel happy (81%), and stay alert in class (76%). However, data on the number of walks to and from school, which was measured using swipecard tracking technology, suggested that the prevalence of children walking to school declined during the 4-week intervention from 29% at week 1 to 12% at week 4. A limitation of this study however, was that it did not include a control school or collect an objective measure of physical activity levels and, given the mixed findings highlighted in that work, further research is needed to better understand the impact of Beat the Street on activity levels longer term.

In this pilot study, we quantitatively evaluate the impact of Beat the Street on levels of active travel using objective measures of change in physical activity recorded by accelerometry. Whilst Beat the Street adopts a whole-community approach that is aimed at increasing active travel within entire neighbourhoods, we focus on the impact of the intervention on active travel to school in a sample of children in the city of Norwich, UK.

## 2 Methods

### 2.1 Intervention

Beat the Street took place within the city of Norwich, UK, for 9 weeks during May-July 2014 and the present study focuses on this scheme. The intervention was restricted to three neighbourhoods located in the northeast of the city; Sprowston, Heartsease, and Thorpe St Andrew, which together covered an area of approximately 5.7 km^2^. In total 40 Beat Boxes were installed in the street environment; 38 were placed on lampposts in the three intervention neighbourhoods and an additional 2 were placed in the city centre approximately 3 km away. One of the aims of Beat the Street was to encourage children to actively travel to school, with the premise that children walking or cycling would find it easier to touch their smartcards on sensors than those using motorised transport. Notably the scheme encouraged children who lived too far from school to be able to walk the entire length of their journey, to take part by either asking their parents to stop the car further from the school gate and walk the remaining part of the journey, or by getting off the bus a stop or two earlier to walk.

Participants were awarded a point each time they touched their smartcard on a sensor, allowing children to compete against other pupils at their school to see who could achieve the most points. Furthermore, the distances between sensors were computed to provide estimates of distances walked and cycled and a target was set for all participating children to “walk and cycle around the world” during the 9 week programme. There was also a wider competition where schools competed against each other, with the winning school at the end of the 9 weeks receiving funding to spend on sports equipment, books, or resources. Schools were also able to compete against other groups taking part in Beat the Street, such as local workplaces, for weekly spot prizes donated by local businesses. A series of promotional events took place regularly while the intervention was running to promote interest in the scheme and encourage participation.

### 2.2 Study design

This study was a pilot non-randomised controlled evaluation of a 9 week intervention. The evaluation was designed using a logic model for the intervention (Table 1) which was developed in collaboration with staff at Intelligent Health, the organisation that designed and implemented Beat the Street. We were guided by CONSORT and STROBE guidelines for reporting of methods and results. Ethical approval was obtained from the Research Ethics Committee at the University of East Anglia prior to the study commencing.

### 2.3 Sample population and recruitment

Children were recruited to take part in the evaluation via two schools; a primary school in the intervention area, plus a control primary school located 7.5 km away on the opposite side of the city, chosen in order to minimise contamination from the intervention. The Headteacher at each school was contacted via a letter and follow-up call to invite their school to participate in the study. Once approval had been obtained, all children in Years 4 and 5 (aged 8–10 years) at both schools were invited to take part. Each child was given an information sheet for themselves, one for their parents, and a consent form in a take-home pack. Children were encouraged to discuss the study with their parents and return the jointly signed consent form within a week. In total, 150 children were invited at the intervention school with 51 (34.0%) agreeing to participate, whereas 56 children were invited at the control school with 29 (51.8%) consenting to take part. Upon completion of the study each child received a certificate and a Frisbee to thank them for their participation.

### 2.4 Data collection

Demographic characteristics including the child’s gender, school they attended, and their school year were collected at the start of the study when children completed their consent form. The children were then measured at three time points including baseline (Week 0, May 2014), during the intervention (Week 7, July 2014), and post-intervention (Week 20, October 2014). The baseline and mid-intervention measurements took place during the school summer term, whereas the post-intervention measurements took place in the autumn term following the long (6 week) school summer vacation. At each of the three measurement times the children were asked to wear an ActiGraph GT1M accelerometer for 7 days. This recorded their activity levels using a 10 second epoch. The children were also asked to complete a simple travel diary that recorded their mode of travel to and from school on the five school days during which measurement took place. This form of travel diary has been validated elsewhere and has been found to provide high convergent validity when compared to GPS data on children’s journeys to school (Oliver et al., 2014). Whilst our primary analysis was based on intention-to-treat, to obtain a per-protocol measure of engagement with the intervention we identified the number of times that each of our study participants at the intervention school touched a Beat Box with their smartcard.

### 2.5 Analysis

All 80 children who took part in the study were included in the analysis of baseline data. However, there was some missing data for the mid-intervention and post-intervention measurement stages. This was due to 4 children dropping out of the study at mid-intervention but later re-joining for the final follow-up, while 2 further children did not complete the final follow-up. In addition, 8 children failed to return their accelerometer at one of the measurement stages. This left 71 children who provided mid-intervention data (50 at the intervention school and 21 at the control), and 75 children who provided post-intervention data (47 intervention children and 28 control).

For the purpose of this analysis we were interested in physical activity measured on school days and in the periods during which the children were likely to be outdoors but not at school, as these were the periods targeted by the school component of the intervention. These included the commute to school period in the morning (8–9 a.m.) and the commute home from school period in the afternoon (3–4 p.m.). We also examined changes in physical activity during the evening (4–10 p.m.) and at weekends (8 a.m. to 10 p.m.). We did this because it has been hypothesised that children might compensate for higher levels of physical activity accrued during the school day, such as those associated with a change from passive to active travel to school, by being less active during evenings and weekends (Frémeaux et al., 2011).

### 2.6 Measurement of change between baseline and follow-up

In order to measure change in physical activity between baseline, mid-intervention, and post-intervention, two physical activity measures were computed. The first was accelerometer average counts per minute (CPM), which measures change in velocity and provides a measure of children’s overall activity levels, including light activity such as walking to school at a slow pace (Robertson et al., 2011). The second was time spent in moderate-to-vigorous physical activity (MVPA), which gave a measure of higher intensity physical activity such as walking to school at a fast pace. MVPA was studied in addition to CPM because higher intensity physical activity is thought to provide additional health benefits, such as improved cardiorespiratory fitness and lower blood pressure and glucose levels (Wenger and Bell, 1986, Foulds et al., 2014, Ross et al., 2015). To calculate the number of minutes each child spent undertaking MVPA each accelerometer data point was classified into one of 4 intensity categories: sedentary (equivalent to≤100 counts per minute (CPM)), light (101–1999 CPM), moderate (2000–3999 CPM), or vigorous activity (≥4000 CPM) (Ekelund et al., 2004) and the relevant data points were then selected for analysis.

For each of these two physical activity measures (average CPM and total MVPA minutes), activity levels recorded at mid-intervention for each of the four time periods (commute to school, commute home, evening after school, and weekend) were subtracted from that recorded at baseline to give change in activity levels between baseline and mid-intervention. Similarly, change in physical activity between baseline and post-intervention was calculated in the same way. These variables were then used as the primary outcome variables for analysis.

Change in mode of travel to school was assessed by comparing the mode children reported using at baseline with that provided at follow-up. Each journey reported in the children’s travel diaries was first classified into whether the child had actively travelled to school (walked or cycled) or passively travelled (taken the car or bus). Each child reported up to 10 journeys for each week they were sampled, which consisted of up to 5 journeys to school and 5 journeys from school. This information was then used to compute the percentage of journeys, based on the number reported, that each child had actively travelled at each of the three measurement periods. This data was then used to compute the change in the percentage of journeys undertaken by active travel at baseline compared to mid-intervention, and also for baseline compared to post-intervention.

### 2.7 Statistical modelling

Statistics describing the sample characteristics were generated for the intervention and control school, and checks for differences between the schools were undertaken using Chi-squared tests. Descriptive statistics were also produced for baseline, mid-intervention, and post-intervention physical activity levels and travel mode, plus change in these outcomes across the study. Here differences between the schools were tested using either an Independent Samples *T*-test or a Mann-Whitney *U* test depending on whether the variable being tested followed a normal distribution.

The association between changes in physical activity between baseline and post-intervention with exposure to the intervention were examined by the use of multiple regression models. Because the outcome variables for change in physical activity levels were found to follow a normal distribution, Ordinary Least Squares (OLS) models were used. In each model the outcome was the absolute change in counts per minute between baseline and post-intervention, with the baseline value being fitted as a covariate. In addition, change in accelerometer wear time between baseline and post-intervention, along with wear time at baseline, was added to the models as a potential confounder to control for the fact that recorded activity intensity may be associated with wear time. Gender and school year were also adjusted for.

Whilst including all the variables mentioned above, two sets of models were fitted which were as follows. The first was based on intention-to-treat, and included all children with a dummy variable being fitted, which differentiated intervention from control school, to determine the intervention effect. The second was based on per-protocol. Again it included all children along with the dummy variable to differentiate intervention from control school but it also included an extra variable that represented engagement with the intervention. This variable was the total number of days on which each child touched a Beat Box, where children who attended the control school were recorded as having engaged on 0 days. The intention-to-treat and per-protocol models were each fitted separately for the four time periods across the week including the weekday commute to school, commute home, evening after school (with an additional model covering these three time periods combined), and weekend. In order to limit the problems of multiple testing, these models focussed on changes between baseline and post-intervention follow-up only. Finally, in addition to fitting models where the outcome was absolute change in CPM, we also fitted similar models for absolute change in MVPA minutes between baseline and post-intervention. All statistical analyses were undertaken in SPSS v22.

## 3 Results

In terms of the characteristics of the sample there were no statistically significant differences between the intervention and control school in terms of the gender of participants or their age, although children at the intervention school were more likely to be female (62.7% versus 41.4% at the control, p=0.065) and in Year 4 (66.7% versus 44.8% at the control, p=0.056).

In terms of engagement with the intervention, 84.3% (n=43) of the children we sampled at our intervention school took part in Beat the Street (i.e. they touched a Beat Box at least once). On average the children touched a Beat Box on 14.5 days (95%CI 10.0, 18.9) and made a total of 78.4 swipes per child (95%CI 37.6, 119.3) across the 9 weeks that the intervention took place. This equates on average to 5.4 swipes per child on each of the days they touched a Beat Box, suggesting that children swiped multiple Beat Boxes per journey.

Table 2 shows unadjusted physical activity and travel mode at baseline, mid-intervention, and post-intervention, plus change in these across the study. At baseline there was no difference in total physical activity measured as CPM or higher intensity physical activity measured as MVPA minutes between the schools. The sample were reasonably active with the children at both schools almost achieving the recommended guidelines for physical activity of 60 minutes MVPA per day. Children at the intervention school reported fewer active travel journeys per week at baseline (median 55%) compared to the control (82.9%), although this difference was not statistically significant (p=0.172).

In terms of change in physical activity across the study, Table 2 shows that there was no difference between the schools with respect to change in CPM (p=0.823), with the control school showing a small decline of −8.3 CPM per child per day between baseline and post-intervention follow-up, while the intervention school showed a similar decline of −10.7 CPM. However, in terms of higher intensity physical activity, whilst both schools showed a decline in MVPA minutes across the study, this decline was significantly smaller at the intervention school (−15.1 min) between baseline and post-intervention versus −23.3 min at the control (p=0.020).

With respect to travel mode to school, Table 2 shows there was no statistically significant difference observed in reported active travel journeys between baseline and mid-intervention follow-up (p=0.328), with both intervention and control schools showing an increase in active travel. However, we found that between baseline and post-intervention active travel increased at the intervention school (+10.0% per child per week, equivalent to 1 extra active travel journey out of a possible 10), while it decreased at the control school (−7.0%), although the difference fell just short of statistical significance (p=0.056). We also found that the percentage of children who had either maintained or increased frequency of active travel commutes to school post-intervention compared to baseline was greater at the intervention school (66.7% of children) than at the control (55.6%). Further, at the intervention school 4 children had switched from a non-active mode of travel at baseline to an active mode post-intervention (25.0% of non-active travellers at baseline), whereas at the control school none of the non-active travellers made this switch.

Table 3 shows the intervention effects from the intention-to-treat models of change in CPM and MVPA between baseline and post-intervention after adjusting for child gender, school year, baseline physical activity, and accelerometer wear time. Here separate models were fitted for weekdays versus weekends and for different times of day. The only statistically significant intervention effect was a decline in weekday evening MVPA of 6.5 minutes amongst intervention compared to control school children. Table 3 also shows the results for the adjusted per-protocol models, with the only statistically significant intervention effect again being a decline of 7 minutes in weekday evening MVPA amongst intervention compared to control children. There was however, evidence of statistically significant associations with engagement, with a positive association between MVPA measured during the morning and afternoon school commute periods and the total number of days across the intervention on which children touched a Beat Box with their smartcard.

Table 3 shows that in terms of the morning commute to school, every day a child touched a Beat Box corresponded to an additional increase in MVPA of 8.3 seconds (0.14 min) on average during the morning commute hour (p=0.001). This equates to an extra 120.4 seconds (2.01 min) of MVPA each day during the morning commute to school for children who touched a Beat Box on 14.5 days (which was the average number of days that children engaged) compared to children with no engagement, and an extra 5.95 min of MVPA each day for the child who touched a Beat Box on the greatest number of days (43 days in this sample). Similarly, in terms of the afternoon commute hour, every day a child touched a Beat Box corresponded to an additional increase in MVPA of 6.0 seconds (0.10 min) on average during the afternoon commute period (p=0.030). This equates to an extra 87.0 seconds (1.45 min) of MVPA each day during the afternoon commute hour for children who touched a Beat Box on a mean number of days (14.5 days), compared to children with no engagement, and an extra 4.30 min for the child who touched a Beat Box on the greatest number of days (43 days).

## 4 Discussion

This study was undertaken to pilot the methods that could be used to undertake a more comprehensive evaluation of the Beat the Street intervention. The nature of the intervention, in particular the fact that the intellectual property is owned by a commercial company, Intelligent Health, and the intervention is delivered to fee paying local authorities, means that randomisation is not easily achievable. Unlike the previous evaluation by Hunter et al. (2015) we were able to include a control school in our evaluation. We relied on a non-randomised control school in another part of the city. Although the two schools were located 7.5 km away from each other, it is possible that there was some contamination from the intervention at the control school. Further, the fact that we had only two schools meant it was not possible to control for school level effects, and it may be activities undertaken at one or both of the schools were atypical in the period of evaluation, although teachers did not report anything different to normal when asked. An alternative in a larger evaluation may be to have a waiting list control, where some schools act as controls yet receive the intervention at a later point in time. This requires a sample of schools who are willing to wait for the programme, something which may not be popular when funds have been committed. A further limitation of the commercial nature of the intervention was that we had no control over its delivery, although qualitative evaluation undertaken by the local City Council suggested that it appeared to be well received by participants in Norwich.

Our aim had been to recruit a sample of 50 children at both the intervention and control school. Whilst we achieved this at the intervention school, we obtained full data for just 29 children at our control school. We had a short period of time, just 8 weeks, after being informed that Beat the Street would take place in Norwich to design the evaluation, obtain ethical approval, and engage the schools and children. Had more time been available we may have been able to achieve more buy-in, particularly from the control school. Our evaluation was not externally funded, limiting our ability to offer incentives to the schools or children, although we did offer all children a Frisbee for participating in our final follow-up and this led to an improvement in participation compared to the follow-up at 7 weeks. Allowing the children to select a gift from a menu of options may have reduced loss to follow-up further. Furthermore, there were some small differences in our sample with regard to child gender. For example, at the intervention school more girls participated than boys (62.7%) yet only 48.3% of children in our target age group were girls at that school. In contrast, at the control school fewer girls participated than boys (41.4%) whilst 48.0% of our target children at the school were girls. Previous studies that have sampled children within the same age range have demonstrated that girls, those of high socio-economic status, and those of a white ethnic background are more likely to participate in physical activity research (e.g. Van Sluijs et al., 2008).

In common with many pilots, our study was underpowered to detect an intervention effect and our findings with respect to changes in physical activity and commuting mode should be treated with appropriate caution. However, our analysis found that Beat the Street did not significantly impact children’s overall physical activity levels during school commute times. There was evidence though that the intervention had a positive impact on higher intensity physical activity during the school commute. We found that, at the intervention school, higher levels of engagement with the intervention were associated with additional MVPA being accrued during commuting times at 20 week follow-up, which was 2.5 months after Beat the Street had ended. Although this effect was relatively small, e.g. for every day a child touched a Beat Box this represented an additional 8.3 seconds of MVPA in the hour before school and an extra 6.0 seconds in the hour after school, it was statistically significant. Furthermore, most children touched a Beat Box on multiple days so together these coefficients equate on average to an extra 3.46 minutes of daily MVPA per child (consisting of 2.01 and 1.45 min during the morning and afternoon commutes respectively) for children who engaged on the mean number of days (14.5 days), compared to children with no engagement. On average, children at the intervention school undertook 16.4 min of daily MVPA during commute times at baseline, so this would be an equivalent increase of 21.1% by post-intervention. Lastly, there was evidence that children at the intervention school were less active during the evening compared to the controls post-intervention. Previous studies have found that children may compensate for higher levels of physical activity accrued during the school day by being less active during evenings (Frémeaux et al., 2011), which may have been the case here.

We also observed that the prevalence of reported active travel increased at the intervention school between baseline and post-intervention by approximately 10.0%, equivalent to one extra active travel journey per child per week, whereas it decreased at the control school by a similar amount, a difference that approached statistical significance (p=0.056). Notably though, we found that at the intervention school a greater percentage of children either maintained or increased active travel between baseline and post-intervention, or switched from non-active to active travel. This suggests that in addition to encouraging active travellers to undertake more active travel, the intervention may have been successful in encouraging some children to switch from non-active modes to active modes. This is a noteworthy finding because shifts in travel mode are particularly difficult to achieve given that habitual travel modes are resilient to change (Shannon et al., 2006, Ferrer and Ruiz, 2013).

It is noteworthy that our per-protocol analysis showed that engagement with the intervention was relatively low with children on average touching a Beat Box on 14.5 days of the nine weeks (63 days) that Beat the Street took place. It could be that children walked to school on the remaining days but chose not to swipe their smartcard due to the extra burden involved. It is likely though that this low engagement with the intervention is the primary reason that we did not observe a larger increase in children’s physical activity levels both during the intervention and after it had ended.

We found some evidence of a dose-response relationship whereby children that engaged more with the intervention, measured by the number of days on which they touched a Beat Box, showed evidence of statistically significant increases in MVPA during the morning and afternoon commutes despite our small pilot sample. This suggests that Beat the Street may be an effective intervention if the challenge of getting children to engage can be overcome. The reasons that engagement was low are unclear. The intervention was well-promoted with advertisements in the local newspaper and on social media. Additionally schools located in the neighbourhoods where the intervention took place were visited by physical activity officers from the local council to inform teachers and pupils about Beat the Street and encourage them to take part.

The weather was wet during July when the mid-intervention measurements took place, with the region receiving 117% of the rainfall that it usually receives during that month compared to the long-term average (Met Office, 2015). Even though the children at both schools were measured during the same week and were subject to the same weather conditions, it could be that Beat the Street is more effective during dry weather conditions as it relies on children to active travel outdoors. Another potential limitation with the intervention concerns its duration which, in the case of the Norwich programme, was just 9 weeks. It could be that this period was not long enough to lead to longer term behaviour change. Indeed theories of behavioural change suggest that individuals may need to go through a number of stages associated with the formulation and implementation of attitudes and beliefs before actually undertaking changes, and this whole process takes some time (Biener and Abrams, 1991). Evidence from elsewhere shows that the dose of an intervention is a key predictor of behaviour change. For example, a systematic review of eHealth interventions for physical activity and dietary behaviour change showed most participants failed to engage in more than half of the expected eHealth activities, yet those interventions with higher utilisation also had better outcomes (Norman et al., 2007). A further factor that might have limited participation in Beat the Street was that Beat Boxes were confined to three neighbourhoods within the city, with the exception of two that were placed in the city centre. Whilst this had the advantage of allowing us to identify a control school that was unaffected by the intervention, geographically restricting the Beat Boxes is likely to have limited the distance between locations that children playing Beat the Street could have been awarded points for actively travelling to.

It may also be that limitations with our evaluation restricted our ability to detect change. This study was a pilot and hence not statistically powered to detect change. Additionally, whilst our control school was located outside the neighbourhoods within which Beat the Street took place, Norwich is a relatively small city and the control school was just 7.5 km away from the intervention school. As such the possibility of contamination cannot be eliminated. Finally, the Actigraph accelerometer we employed, whilst widely used in studies of children (De Vries et al., 2009) is waist worn and this may have reduced our ability to detect changes in cycling behaviours (Sirard and Russell, 2001). However, relatively few children in our sample (8 children or 10% in total) reported regularly cycling to school and walking was by far the most popular means of active travel, with 52 children (65%) reporting that they walked to school at least once during our three measurement periods. Accelerometers are known to adequately capture time spent walking (Tudor-Locke et al., 2002).

It is noteworthy that Cugelman (2013) warns against the misconception that the employment of gamification tactics in an intervention will inevitably lead to higher levels of engagement. It may be for example that the rewards being offered on this occasion were not enough to engage children or were not meaningful to them. In addition there is evidence that tailored feedback linked to individualised behavioural recommendations are particularly effective (Kreuter et al., 2000), yet this is hard to provide in community level interventions such as this. A further potential problem with Beat the Street was the absence of family support for behaviour change. Indeed O’Donnell (2005) highlights that in order to achieve behaviour change individuals require four components: knowledge, skills, motivation, and opportunity. While Beat the Street provides opportunities for individuals to increase their physical activity by engaging in the intervention and motivation in the form of rewards, it is possible that support is needed to provide the knowledge and skills to initiate active travel behaviours.

## 5 Conclusions

In conclusion we found no evidence of a large intervention effect of Beat the Street at 20 week follow-up, although we did observe that self-reported active travel increased at the intervention school compared to the control. Overall engagement with the intervention, based on the use of smartcards, was low but there was evidence that children who engaged more increased their school commute time physical activity at follow-up. A challenge may therefore be to increase engagement which may substantially improve the effectiveness of the intervention. Based on feedback, the intervention is being adapted to encourage greater family participation, and may therefore form a promising focus for a larger evaluation.

## Competing interests

The authors declare that they have no competing interests.

## Figures and Tables

**Table 1:** Logic model for Beat the Street.

**Programme efficiency**			**Programme effectiveness**		
**Inputs**	**Activities**	**Outputs**	**Anticipated outcomes during intervention**	**Anticipated outcomes after intervention**	**Leading to… (impact)**
• Equipment (Beat Boxes and smartcards) • Efforts to attain engagement (marketing and publicity)	• Install Beat Boxes • Roll out publicity • Sign-up participants • Motivate participants (via competitions and weekly spot prizes) • Monitor programme take-up and use	*Efficiency of recruitment*	• Positive, affirmative experiences of scheme (participants had fun, won prizes, parents of children participating saved money)	• Continued positive, affirmative experiences of walking/cycling	
		• Percentage of target audience signed-up			
		*Efficiency of retention and engagement*			
					• Habitual formation of active travel behaviours • Improved physical activity behaviours
		• Continued participation over the duration of the intervention (total number of days on which participants touch a Beat Box) • Level of engagement (total number of Beat Box touches participants make)			
				***And compared to baseline*:** • Increased active travel and walking for pleasure • Increased physical activity		
			***And compared to baseline:***		
			• Increased active travel and walking for pleasure (as measured by travel diaries) • Increased physical activity due to increased active travel, both overall and moderate-to-vigorous activity (as measured by accelerometery)		

**Note:** Assumes that those in the scheme are not already highly physically active, and are not already regularly using active transport (otherwise outcomes are hard to achieve).

**Table 2:** Unadjusted physical activity (CPM and MVPA minutes) and travel mode to school at baseline, mid-intervention, and post-intervention, plus change in these across the study. Values are mean and 95% CI, unless otherwise stated.

	**Control school**	**Intervention school**	**Difference between schools**
**Physical activity**			
Daily recorded CPM baseline	96.3 (87.2, 105.4)	97.4 (85.8, 109.1)	p=0.876
Daily recorded CPM mid-intervention	98.9 (81.4, 116.4)	88.3 (75.5, 101.0)	p=0.317
Daily recorded CPM post-intervention	87.0 (68.0, 106.0)	87.6 (76.7, 98.6)	p=0.950
Daily recorded MVPA mins baseline	57.2 (50.5, 64.0)	59.5 (52.1, 66.8)	p=0.685
Daily recorded MVPA mins mid-intervention	44.8 (35.7, 53.9)	42.5 (35.6, 49.4)	p=0.683
Daily recorded MVPA mins post-intervention	33.3 (27.8, 38.8)	46.4 (38.9, 53.9)	**p=0.020**
**Change in physical activity**			
Change in recorded CPM between baseline and mid-intervention	+0.1 (−19.2, 19.5)	−9.9 (−26.7, 7.0)	p=0.426
Change in recorded CPM between baseline and post-intervention	−8.3 (−26.4, 9.9)	−10.7 (−23.8, 2.3)	p=0.823
Change in recorded MVPA mins between baseline and mid-intervention	−16.8 (−24.9,−8.7)	−17.1 (−25.1,−9.1)	p=0.683
Change in recorded MVPA mins between baseline and post-intervention	−23.3 (−30.0,−16.6)	−15.1 (−23.2,−6.9)	**p=0.020**
**Travel mode to school**			
Percentage of school commutes baseline that were reported using active travel (median, IQR)	82.9 (25.0, 100.0)	55.0 (7.5, 88.1)	p=0.172
Percentage of school commutes mid-intervention that were reported using active travel (median, IQR)	100.0 (50.0, 100.0)	60.0 (0.0, 100.0)	p=0.052
Percentage of school commutes post-intervention that were reported using active travel (median, IQR)	83.3 (10.0, 100.0)	80.0 (0.0, 100.0)	p=0.859
**Change in travel mode to school**			
Change in % of school commutes reported using active travel between baseline and mid-intervention	+2.5 (−7.8, 12.8)	+8.6 (0.7, 16.4)	p=0.328
Change in % of school commutes reported using active travel between baseline and post-intervention	−7.0 (−22.7, 8.8)	+10.0 (1.6, 18.4)	p=0.056

**Table 3:** Intention-to-treat and per-protocol models for change in physical activity (CPM and MVPA minutes) between baseline and post-intervention. The results are stratified by weekdays versus weekends and by time of day. All values have also been adjusted for gender, school year, baseline physical activity, baseline device wear time, and change in device wear time between baseline and post-intervention.

	**Intention-to-treat**				**Per-protocol**			
	**CPM**		**MVPA mins**		**CPM**		**MVPA mins**	
	**Mean (95% CI)**	**p-value**	**Mean (95% CI)**	**p-value**	**Mean (95% CI)**	**p-value**	**Mean (95% CI)**	**p-value**
**Weekdays all times: 8 a.m. to 10 p.m.**								
Intervention school a	−16.15 (−35.88,3.59)	0.107	−3.40 (−10.09,3.30)	0.315	−14.91 (−36.19,6.38)	0.746	−5.38 (−12.41,1.64)	0.131
Beat Box touch days^b^	–	–	–	–	−0.14 (−0.99,0.72)	0.746	+0.24 (−0.05,0.52)	0.102
**Weekdays morning commute: 8–9 a.m.**								
Intervention school a	+1.00 (−25.34,27.35)	0.940	+0.20 (−1.74,2.15)	0.836	−9.06 (−37.34,19.21)	0.524	−1.13 (−3.08,0.83)	0.254
Beat Box touch days^b^	–	–	–	–	+1.02 (−0.13,2.16)	0.081	+0.14 (0.06,0.22)	**0.001**
**Weekdays afternoon commute: 3–4 p.m.**								
Intervention school a	−7.21 (−37.57,23.14)	0.637	+0.22 (−1.92,2.35)	0.840	−16.71 (−48.37,14.95)	0.295	−0.51 (−2.68,1.66)	0.638
Beat Box touch days^b^	–	–	–	–	+1.17 (−0.13,2.47)	0.078	+0.10 (0.01,0.19)	**0.030**
**Weekdays evening: 4–10 p.m.**								
Intervention school a	−35.51 (−73.91,2.88)	0.069	−6.51 (−10.97,−2.05)	**0.005**	−29.35 (−70.04,11.35)	0.154	−7.03 (−11.77,−2.29)	**0.004**
Beat Box touch days^b^	–	–	–	–	−0.76 (−2.40,0.88)	0.358	+0.07 (−0.13,0.26)	0.503
**Weekends all times: 8 a.m. to 10 p.m.**								
Intervention school a	−2.41 (−52.73,47.91)	0.924	−3.21 (−15.76,9.34)	0.610	−1.38 (−55.08,52.31)	0.959	−3.60 (−17.89,10.68)	0.615
Beat Box touch days^b^	–	–	–	–	−0.57 (−10.13,9.00)	0.906	+0.04 (−0.57,0.64)	0.906

a Whether the child attended the intervention school (no=0, yes=1).

b Total number of days that the child touched a Beat Box with their smartcard.
